# John W. Gadsden

**Published:** 1896-09

**Authors:** 


### NECROLOGY.

JOHN W. GADSDEN, M.R.C.V.S. The sudden termination of the life of this well-known veterinarian at Philadelphia on the evening of August 12, 1896, at the age of sixty-four years, closed the career of one who had earned an enviable and untarnished reputation in the profession among the people who knew him that will live many years after him, and grow brighter and more forceful as time wears on. No higher tribute could any one yearn to win than that which is paid to his lifelong career by friend and foe: "He was honest." Every criticism that lasted for the day or hour during his long career is now silenced by the consent of all in the summing up that "He was honest," and no man's money or influence ever purchased or swayed his opinion in the purchase or sale of a horse. The evildoer, the gyp, the dishonest dealer, feared him to a point of hatred, because of his untarnished reputation. Ever attentive to the needs of his patrons and always ready to respond to their calls, for a long number of years he enjoyed one of the most lucrative private practices in our country, and retired in 1891, with a well-earned competency.

He was a graduate of the London School of the class of 1858, and had served in official capacity in the old country in several of the epizoötics that prevailed, and in the United States he was one of the first to be employed in the control and eradication of contagious pleuro-pneumonia. This proved a very attractive field of work, so much so that even after his retirement from practice he personally travelled many miles, in 1893, to investigate alleged outbreaks of this disease, and presented his conclusions at the First International Veterinary Congress of America, held at Chicago in the same year, challenging those who so reported to produce a single case in the length and breadth of our country. He was the prime mover in breaking up the practice of selling veterinary diplomas in Philadelphia by Dr. McClure, who held the charter and franchises of the old Philadelphia Veterinary College.

For two years he was a member of the United States Veterinary Medical Association, but resigned, as he was no longer in practice. He was a good friend of the Montreal Veterinary

College, and later the Veterinary Department of the McGill University. He was for many years one of the regular examiners of this school, and presented to that institution his library and many of the specimens collected during his long term of practice.

Dr. Gadsden was a prominent member of the St. George Society of Philadelphia, and for two years its President, where he dispensed the freest charity to the deserving needy, and was ever ready to aid the work of this organization in every way within his power.

About a year before his death Dr. Gadsden married the widow of the late Dr. Charles B. Michener, who survives him.

His remains were followed to the grave by a large number of the members of the above-named society and by many veterinarians, among others Drs. Francis Bridge, D. James, W. S. Kooker, Frank Standen, and W. Horace Hoskins.

